# Development and Characterization of a Compact Device for Measuring the Braking Torque of a Vehicle

**DOI:** 10.3390/s20154278

**Published:** 2020-07-31

**Authors:** Ester Olmeda, María Garrosa, Susana Sanz Sánchez, Vicente Díaz

**Affiliations:** 1 Department of Mechanical Engineering, Universidad Carlos III de Madrid, Avda. de la Universidad 30, 28911 Leganés, Spain; mgarrosa@ing.uc3m.es (M.G.); ssanz@ing.uc3m.es (S.S.S.); vdiaz@ing.uc3m.es (V.D.); 2 Institute for Automotive Vehicle Safety (ISVA), Universidad Carlos III de Madrid, Avda. de la Universidad 30, 28911 Leganés, Spain

**Keywords:** braking torque, strain gauge, disc brake, roller brake tester, adherence

## Abstract

In this article, a new force transducer is designed, developed and built for the measurement of braking forces in the wheel rim of a motor vehicle. The parameters of the transducer design are justified using numerical simulation. In order to install it in the vehicle in a simple and interference-free way, the metal base of the caliper rod is used. It is manufactured and installed in a vehicle in order to obtain the signals of the wheel braking torque, in real time, and at different speeds of circulation, carrying out several tests on the track. Subsequently, data are obtained from calculations of the disc brake system itself. The latter provides instantaneous adherence values between the brake pad and the disc.

## 1. Introduction

Comparing the vehicles manufactured in the last 20–25 years with the current ones, there are abundant differences in aspects such as comfort, economy, functionality, and in particular, safety aspects, both in active and passive systems. Active safety systems help prevent traffic accidents and passive systems protect passengers once an accident has occurred. A vehicle’s braking system is one of the most important active safety systems. Braking systems have been improved and electronic systems have been incorporated into them as an aid to vehicle control. However, regardless of the additional electronic systems, braking is still carried out in the same way, by acting on the friction-line brake shoes with pneumatic or hydraulic systems. For example, there has been a great deal of research into the temperature that brakes can withstand [1,2,3] or the characteristics and properties of the materials used [4,5,6]. However, the structure and components of the system have hardly changed.

One of the most common types of braking systems in commercial vehicles today are disc brakes. A disc brake is a braking system in which a disc that rotates in solidarity with the vehicle wheel is subjected to friction by brake pads with a high coefficient of friction. The brake pads are arranged in a part called the caliper, which is in turn fixed solidly to the structure of the car. A hydraulic circuit pushes the brake pads against the disc with sufficient force to transform all or part of the kinetic energy of the vehicle in movement, into heat, until it is stopped or its speed is reduced, as the case may be. 

When the driver presses on the brake pedal, the pedal pushes a master cylinder that delivers brake fluid to the hydraulic brake circuit at a certain pressure. The hydraulic circuit sends the brake fluid to the cylinders housed in the caliper. There, pistons move and press the brake pads in an axial direction on both sides of the brake disc attached to the wheel. The fluid is distributed at the same pressure throughout the circuit to each of the pistons on each wheel, either to the front discs or to the rear discs or drums, depending on the system of the rear axle. Depending on the pressure of the fluid and the section of the cylinders, the braking force will vary on the surface of the disc. 

For the safety of a motor vehicle, it would be very important to know the braking force on the wheel in real time. There are some indirect methods of measuring the braking force [7,8] and others that required a transformation of several vehicle systems [9]. However, at present, there is no device that allows this parameter, or the braking torque, to be measured directly without modifying the vehicle.

Studies on friction brakes have been conducted, mostly through classical mathematical formulations and experiments with samples or field tests in a stand. The classical formulations, related to the study of the main dynamic aspects of the brakes, are mainly based on rotational calculation models. These models are based on a series of assumptions/simplifications, including [10]:

- The braking force instantly reaches its steady-state value;
- The space (gap) between the friction lining and the disc/drum is neglected;
- There are no abnormalities in the kinematic chain—the disc/drum does not have deviations from roundness nor run-out (radial or lateral).

More recently, the capability to conduct further research on the dynamic performance of braking systems through computer models and simulations, based on CAE tools, such as the finite element method (FEM), multi-body dynamics (MBD), etc., has been improved. The operation and performance of automotive disc and drum brakes has been studied with these models. Using FEM, different problems have been analyzed. These include, for instance: the thermomechanical behavior of the dry contact between the disc and the brake pads during the braking phase base and the following of the evolution of the global temperatures by a numerical modeling using ANSYS [11,12], the effects of different parameters on mode-coupling instability [13], the optimization of the critical design parameters of the magnetic circuit of a magnetorheological brake [14], etc.

These models help represent and model the reality, and they allow us to identify how the systems would behave. However, the models must be checked against experimental measures and the hypotheses on which they are based must be confirmed. Some of these models allow us to estimate the braking force and the adherence coefficient between the brake pad and the disc (which is known in some studies as the brake linings’ coefficient of friction (BLCF)). The latter magnitude is of great importance to monitor the brake operation and increase the performance of control systems such as ABS, TC and ESP. In most studies it is considered constant, without taking into account its dependence on temperature; fading; bedding; hysteresis against the pressure; hysteresis against the speed, wear and aging; variation in the environmental conditions; and the chemical composition and mechanical properties of each component [15,16].

Therefore, the direct determination, via measurement, of the braking force and the adherence coefficient between the brake pad and the disc would be of great interest. 

However, the only way that currently exists to measure the braking force in a vehicle is the brake tester. A brake tester is a measuring instrument used to evaluate and measure the longitudinal braking force on the wheel(s) of the same axle. There are flat and roller measurement benches [17], the latter being the most widely used, for example, in periodic roadworthiness testing. 

**Figure 1:**
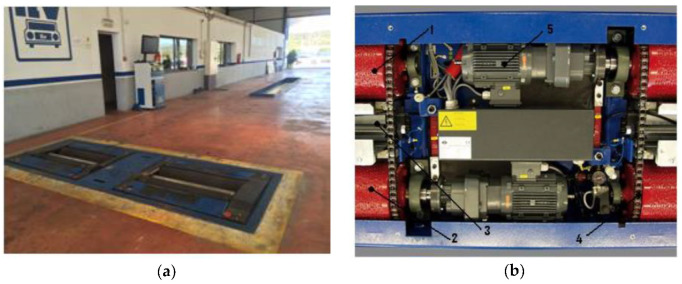
Roller brake tester. (**a**) General view and (**b**) main elements.

The system consists of a roller bed and a control computer that displays the results. Figure 1a shows a roller brake tester: the bed and the computer. This type of bed is used in workshops and vehicle inspection stations. Figure 1b shows the components of a roller brake tester. The main elements that make up the bed are:

- Four pinch rollers (1, 2 in Figure 1b)
- Two feeler rolls (3 in Figure 1b)
- Two motors and strain gauges (5 in Figure 1b)
- Drive chains connecting the pair of drive rollers on each side (4 in Figure 1b)

In order to verify the correct functioning of the brake system, these inspections check the efficiency of the brakes, the ovality and the unbalance, all of which are quantifiable values obtained by this device.

This equipment, however, is influenced by various factors and has limitations and errors [18,19,20,21,22]. Furthermore, it has been shown that it can give different test results depending on the model of brake tester used [23,24].In addition, the roller size and the tire conditions showed a significant influence on the test results [25,26]. Finally, in the case of heavy vehicles the braking efficiency of the vehicle, which is evaluated by measuring the braking forces on the roller brake tester, also depends on the vehicle load [19,20]. In this case, developing procedures to inspect vehicles in a fully laden condition, with methods of pressure measurement or with equivalent methods have been the main difficulties [27].

The main disadvantages of this system are the following:

- It is not designed to take into account the influence of load transfer to the front axle that occurs during braking under driving conditions.
- Aerodynamic effects cannot be taken into account.
- It cannot check the anti-lock braking system (ABS) that most cars have today because the system operates by measuring the rotation of all four wheels of the car simultaneously or because the test speed is too low.

Within this framework, this article develops an autonomous device with respect to the vehicle’s own brake system (i.e., without interfering with the correct functioning of the vehicle). It consists of a system capable of characterizing the braking of a vehicle based on a measurement sensor that allows knowing the force existing in the brake disc when it is activated by the driver through the brake circuit. The sensor is located in the brake caliper of a disc brake. The data of the deformation suffered in the fixing rod of the brake caliper jaw will be used. This is a new device specially designed to measure the braking torque of a vehicle.

Therefore, a device will be designed and built to measure the deformation that the brake system caliper attachment rod undergoes when the vehicle brakes.

**Figure 2:**
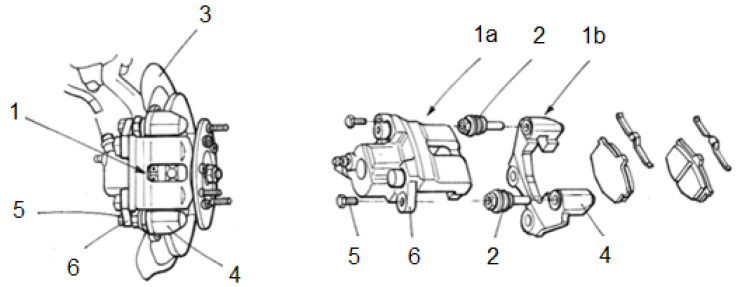
Main mechanical parts of a floating caliper disc brake system.

Figure 2 shows the main mechanical parts of a disc brake system. In the vehicle, the clamp (1) is fixed by means of a pair of rods (2) that connect a fixed part of the clamp (1a) with a movable part of the clamp (1b), so that the movable part can slide in a direction perpendicular to the brake disc (3) in order to allow the brake pads to be centred around the brake disc. More specifically, one end of each rod is inserted into a hole in a portion (4) of the moving part of the caliper. A fixing screw (5) in turn passes through a through hole in a portion (6) of the fixed part of the clamp and is threaded into a longitudinal hole in the other end of the rod. Thus, the two parts of the clamp are connected in a sliding manner and are automatically centered when the driver operates the brake pedal (see Figure 2).

The mechanical stresses generated during vehicle standstill are absorbed by these rods, which consequently deform elastically during the braking process. It is intended that the sensor measures this deformation, which is proportional to the stresses that appear during braking and, therefore, it is also proportional to the braking force applied.

## 2. Materials and Methods

**Figure 3:**
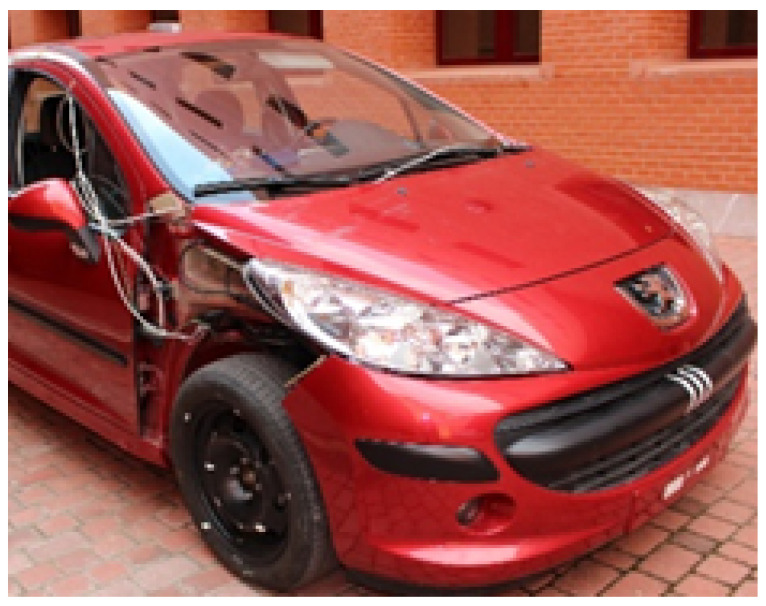
Peugeot 207 1.6HDI 16v.

**Figure 4:**
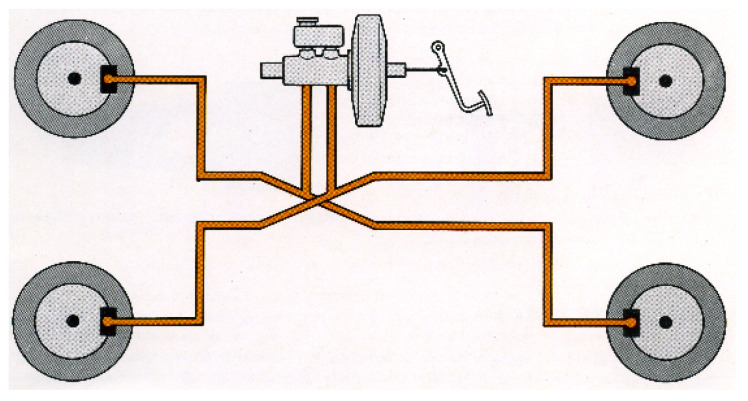
X-brake circuit.

The vehicle chosen for the experimental tests and the installation of the transducer is a commercial Peugeot 207 1.6 HDI 16v diesel-powered car (see Figure 3). It has a 4-wheel hydraulic x-type brake circuit (see Figure 4).

In this work, a strain device is designed by instrumentation on one of the brake caliper rods. This allows measuring the braking force independently of the configuration of the brake circuit, since it is directly associated to the wheel hub. In order to test its effectiveness, a prototype has been built and subsequently implemented in a passenger car.

The caliper rods of a disc brake system allow the pads to be moved laterally, in a direction perpendicular to the rotation of the disc. This movement, caused by two brake cylinders activated by the pressure of the hydraulic circuit, causes the pads to compress the brake disc itself. Given the adherence of the pads, the disc stops and, therefore, the vehicle’s wheel is braked. Pad oscillation depends on the chosen disc brake system (there are mainly four types of brake calipers: fixed caliper, Girling swing caliper brake, Girling sliding caliper brake and Lockheed swing caliper brake. The substantial difference between them is that they are operated by one or two brake cylinders and some of them move sideways with an oscillation with respect to the plane containing them).

In either case, the caliper rods remain attached to the wheel hub, providing a support point at all times.

The design of the transducer itself consists of significantly lowering the area of greatest rod deformation. The strain gauge is attached to this high strain zone. This methodology has been used because it is impossible to incorporate a force transducer due to the limited space available. In this way, the operation of the brake is not interfered with at all.

### Installation of the Device

The strain gauge used has the characteristics shown in Table 1.

**Figure 5:**
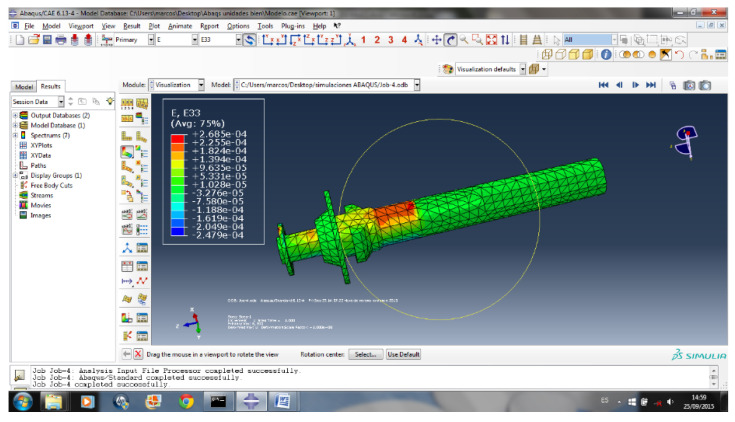
Results of the FEM simulation [Source: ABAQUS 6.13-4].

Before the sensor is manufactured, its operation is checked numerically. For this purpose, a simulation of the part is carried out by means of FEM in Abaqus (Figure 5) to determine the area where the microdeformations are greater to carry out the installation of the strain gauge. In the simulation, loads are applied in the points where, once the sensor is installed, it will support the efforts and a stress analysis is carried out.

**Figure 6:**
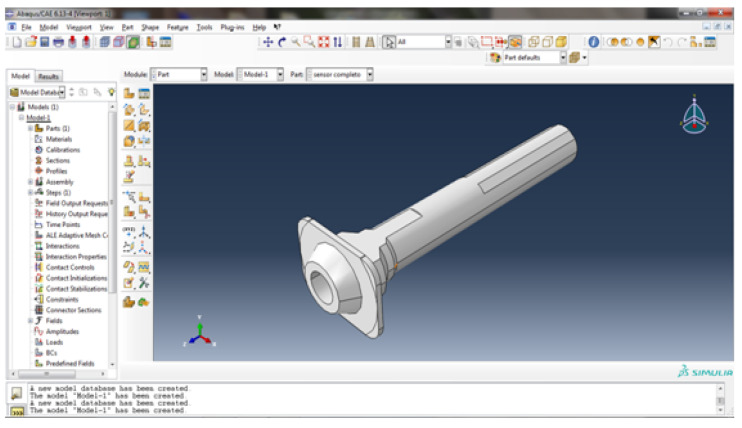
Machined rod [ABAQUS Own Source 6.13-4].

The numerical simulation allows the location of the gauge to be precisely determined. Once it is determined, the rod is machined and the gauge is glued. By weakening the caliper rod slightly, the sensitivity of the sensor is increased (Figure 6). In this case, the area chosen is that between the drive supports of the jaw itself (area protected by the dust cap).

**Figure 7:**
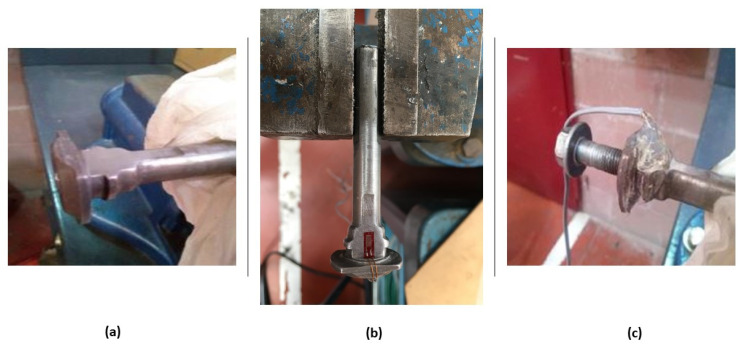
(**a**) Machining, (**b**) gluing, and (**c**) hot-melt glue coating.

Once the area has been machined and sanded, it is important to remove dirt with alcohol to promote the bonding of the sensor and avoid problems during the tests. Cyanoacrylate is used to bond the gauge due to the mechanical characteristics and durability it offers over time. Finally, the whole system is covered with hot-melt glue, avoiding the accumulation of dirt on the surface and possible undesired contact between the different elements involved (metal-to-metal contact) (see Figure 7).

**Figure 8:**
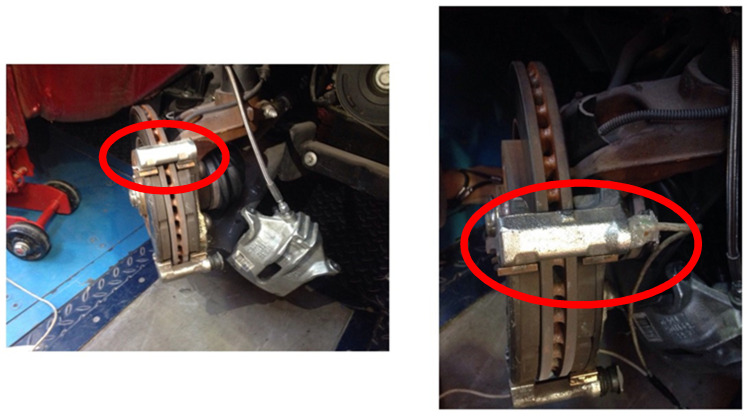
Rod caliper installation in the vehicle’s braking system. (**left**) General view (position) and (**right**) detail.

Once the sensor is glued, the rod is installed in its original location in the vehicle’s braking system (see Figure 8). It shows the area of application of stress during braking.

## 3. Results

### 3.1. Device Calibration 

In order to determine the wheel braking torque, it is necessary to calibrate the new device, to determine its behaviour.

**Figure 9:**
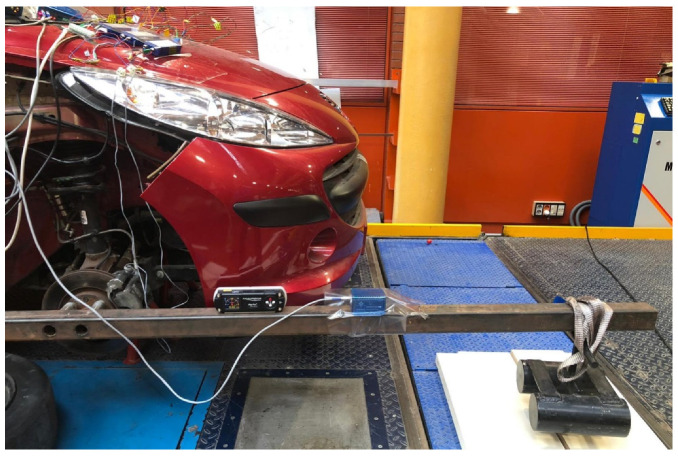
Bar bolted to the rim wheel for the device calibration.

A steel bar, designed to act as a lever, has been used in the calibration. To generate a controlled torque, a calibrated mass is used which moves along the bar. Specifically, the chosen (calibrated) mass has a value of 30 kg, and it is placed at points that are 1 and 1.5 m from the wheel’s center of rotation (see Figure 9). This device makes it possible to obtain the relationship between the voltage shown on the deformation sensor and the resulting braking torque exerted. 

Once the calibration system has been installed, the driver of the vehicle must operate the brake pedal until the bar is horizontal and static. The horizontality is checked by means of an electronic inclinometer. Gradually, the brake pedal shall be released until the torque generated on the wheel is greater than the pressure applied in the brake circuit, producing the rotation of the wheel. At this point, the value indicated by the strain sensor is recorded.

In order to know when the rotation of the wheel takes place, an inertial measurement unit is placed on the bar, which will be synchronized with the data acquisition system.

**Figure 10:**
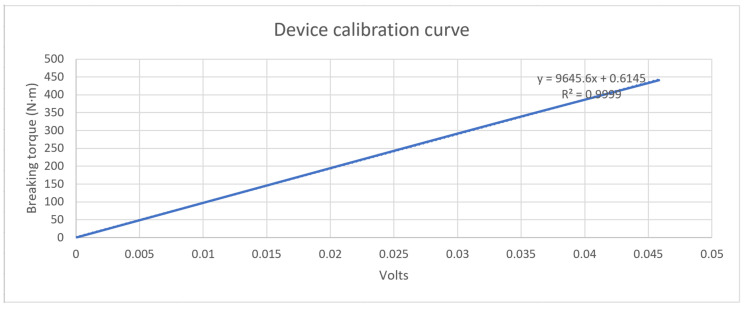
Device calibration curve.

Figure 10 shows the calibration curve obtained for the device located on the right front wheel brake caliper clamp fixing rod. 

The calibration curve is as follows:

Braking torque (N·m) = 9645.6·V + 0.6145(1)

### 3.2. Track Tests

**Figure 11:**
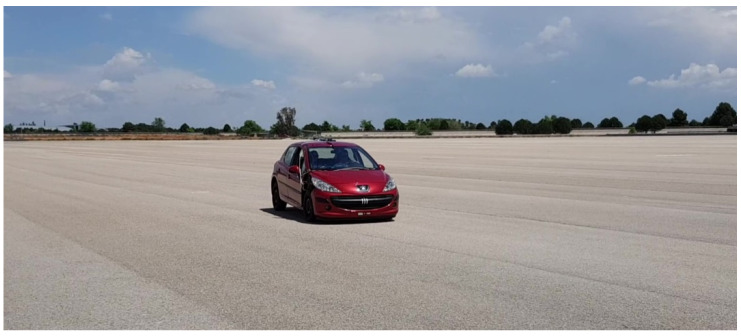
Track tests.

Track tests were carried out to check the correct operation of the new on-board device (see Figure 11).

A progressive braking is performed, which is characterized by increasing in a linear way in time. The slope of this breaking curve determines the degree of severity of the braking. Tests have been carried out in this way until the vehicle is stopped. Pressing the brake pedal gradually makes the pistons and pads position themselves correctly against the brake disc. This type of braking is the one that is usually done when driving a vehicle. It also coincides with the one performed in the brake tester test.

The tests were conducted at speeds of 20 km/h, 30 km/h, 40 km/h, 50 km/h, 60 km/h, 70 km/h and 80 km/h. As each driver tends to brake differently, the tests were conducted by 14 different drivers.

**Figure 12:**
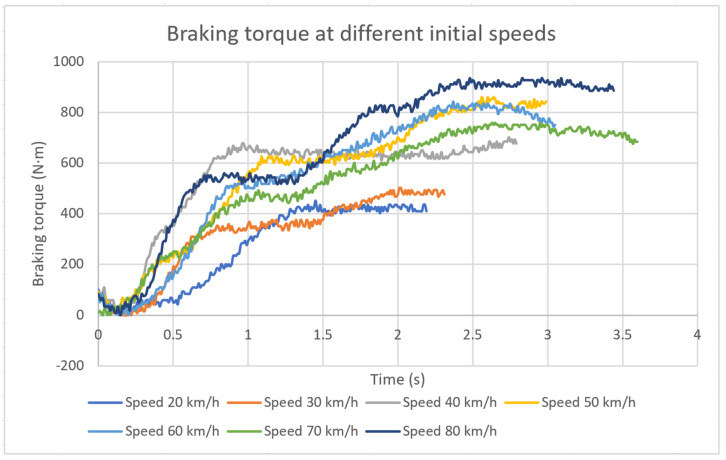
Braking torque obtained during the track test with the proposed device at different initial speeds.

The following example shows the curves obtained using the torque transducer designed in this article in a single-conductor test (Figure 12). The obtained calibration curve (1) was used to convert the electrical signal into units of torque. The graph shows the braking torque obtained for different speeds where braking starts.

The figure shows that, as the speed at which braking starts to increases, the time needed to stop the vehicle also increases. Likewise, the torque applied on the brake increases, even doubling this value, from 20 km/h to 80 km/h.

The braking force measured by the proposed device can be calculated from the braking torque. To save resources, only one rod is used. Assuming that both rods would have the same measurement, the braking torque will be twice as much as that measured with only one of them.

(2) $$N=2\cdot F_{d}\cdot r$$

Being:

*N*: Braking torque
*F_d_*: Force on device
*r*: Distance from the centre of the wheel to the point where the force is applied (point of application of the resulting force on the brake pad)

**Figure 13:**
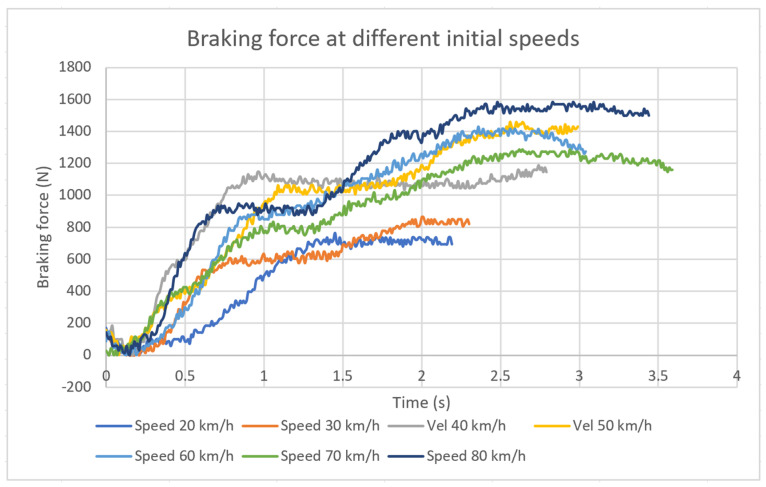
Braking force on the front axle at different speeds.

Applying the expression (2) gives the value of the braking force in the device (Figure 13).

From the data obtained it is also possible to calculate the adherence of the pads used in the braking. 

Taking into account the hydraulic circuit, the force acting on each pad (T) will be given by:

(3) $$T=\frac{\pi \cdot d^{2}}{4}\cdot p_{h}$$

where d is the diameter of the hydraulic cylinder and $p_{h}\;$the hydraulic pressure.

Assuming that the pressure distribution, *p*, exerted by the pad on the disc is constant over the entire contact surface, it is verified that:

(4) $$p=\frac{T}{S_{pad}}$$

To calculate the surface area of the pad ($S_{pad})$ a differential element of the area of the pad is obtained, which is given by the following expression:

(5) dS=r·dθ·dr

Integrating along the whole surface is obtained:

(6) $$S_{pastilla}=\int_{0}^{\alpha }\int_{R_{i}}^{R_{e}}r\cdot dr\cdot d\theta =\frac{\alpha \cdot \left(R_{e}^{2}-R_{i}^{2}\right)}{2}$$

where $R_{e}$ and $R_{i}\;$represent respectively the exterior and interior radius of the brake pad and α represents the angle covered by it. Taking into account the above expressions, the pressure exerted will be:

(7) $$p=\frac{\frac{\pi \cdot d^{2}}{4}\cdot p_{h}}{\frac{\alpha \cdot \left(R_{e}^{2}-R_{i}^{2}\right)}{2}}=\frac{\pi \cdot d^{2}\cdot p_{h}}{2\alpha \cdot \left(R_{e}^{2}-R_{i}^{2}\right)}$$

On each differential surface element, dS, a differential normal force acts on the pad, $dF_{n}$:

(8) $$dF_{n}=p\cdot dS=p\cdot r\cdot d\theta \cdot dr$$

(9) $$F_{n}=\int_{0}^{\alpha }\int_{R_{i}}^{R_{e}}p\cdot r\cdot d\theta \cdot dr$$

(10) $$F_{n}=\frac{1}{2}p\cdot \alpha \cdot \left(R_{e}^{2}-R_{i}^{2}\right)$$

The normal force between the pads and the disc generates a frictional force (*F_t_*) (μ represents the coefficient of friction between the pad and the disc) such that:

(11) $$dF_{T}=\mu \cdot dF_{n}=\mu \cdot p\cdot dS=\mu \cdot p\cdot r\cdot d\theta \cdot dr$$

(12) $$F_{T}=\int_{0}^{\alpha }\int_{R_{i}}^{R_{e}}\mu \cdot p\cdot \;r\cdot dr\cdot d\theta =\frac{1}{2}\alpha \cdot \mu \cdot p\cdot \left(R_{e}^{2}-R_{i}^{2}\right)$$

This force depends on its geometrical dimensions, the specific pressure *p* between the pad and the disc (calculated above), and the friction between the two.

The friction force generates a torque with respect to the centre of rotation of the disc:

(13) $$dN=r\cdot dF_{T}=r\cdot \mu \cdot p\cdot dS=r^{2}\cdot \mu \cdot p\cdot dr\cdot d\theta$$

By integrating, the braking torque generated by a single brake pad is obtained:

(14) $$N=\int_{0}^{\alpha }\int_{R_{i}}^{R_{e}}\mu \cdot p\cdot r^{2}\cdot dr\cdot d\theta =\frac{\alpha \cdot \mu \cdot p}{3}\left(R_{e}^{3}-R_{i}^{3}\right)$$

For the disk, i.e., taking into account two pads (one on each side), the total torque generated is:

(15) $$N_{disco}=\frac{2\cdot \alpha \cdot \mu \cdot p}{3}\left(R_{e}^{3}-R_{i}^{3}\right)$$

Clearing the adherence coefficient between the pad and disc:

(16) $$\mu_{p}=\frac{3\cdot N}{2\cdot \alpha \cdot p\cdot \left(R_{e}^{3}-R_{i}^{3}\right)}$$

As an example, the calculations for the speed of 50 km/h will be performed. The brake characteristics of the vehicle being tested are shown in Table 2.

**Figure 14:**
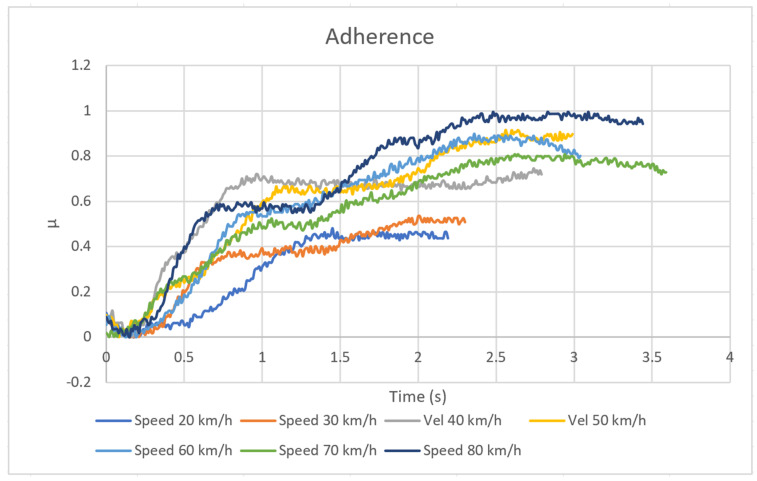
Coefficients of adherence used for different speeds.

Knowing this data and the hydraulic pressure, from the expression (7) the pressure exerted by the pad on the disc is calculated. Knowing this value of *p* and the value of the braking torque (N) measured at each instant (Figure 12) (calculated from the new device), from the expression (16) the adherence coefficient used at each instant is obtained. The result is shown in Figure 14.

Figure 14 shows that the higher the driving speed, the greater the braking efficiency and therefore the driver makes more use of the adherence between the pad and the disc.

## 4. Discussion

As discussed in Section 1, the only direct way that is currently available to measure the braking force on a vehicle is the brake tester. However, this equipment is influenced by several factors and has limitations and errors. In this article, a new force transducer is designed, developed and built to measure the braking forces in the wheel of a motor vehicle, independent of the vehicle’s own braking system (i.e., without interfering with the correct functioning of the vehicle). It is a system that is capable of characterizing the braking of a vehicle from a measurement sensor that allows knowing the force existing in the brake disc when it is activated by the driver through the braking circuit. The sensor is located in the brake caliper of a brake disc.

The operation of this new device is essentially as follows. First, a strain transducer (strain gauge) is installed in the described position (Section 2) of the clamping rod. Then, the necessary empirical tests are carried out to determine the correspondence between caliper rod strain and wheel braking torque, and the device is calibrated as described in Section 3.1. The correspondence ratio is checked to be linear. 

This relationship allows the strain gauge to transform the strain data into an electronic signal in real time. Through the calibration function these data are expressed as wheel braking force data. These brake force data can be used later in different ways, some of which are described in the conclusions section.

The main advantages of the new device compared to the existing methods are listed in the following Section 5.

In future works, it would be interesting to analyze in depth the stability of the device in varying conditions. For example, it would be interesting to analyze the spread of the braking torque versus the output voltage over time and under different environmental conditions. Among the environmental conditions, it would be especially interesting to analyze the influence of temperature, humidity and wind speed.

## 5. Conclusions

A new system is presented that is capable of characterizing the braking of a vehicle based on a measurement sensor that allows the braking force on the wheel to be known when it is actuated by the driver through the brake circuit. For this purpose, data on the deformation suffered by the brake caliper fixing rod will be used. The sensor is located in the brake caliper of a disc brake. It is a new device specially designed to measure the braking torque of a vehicle.

Until now the only way to directly measure the braking force in a vehicle was the brake tester. In any case, this measurement is done on a static bench, without any load transfer, making mistakes, as mentioned above.

The main advantages of the new system over existing methods are as follows:

- As it is integrated into the brake system itself, it can carry out measurements continuously and under real conditions.
- It takes into account the influence of the load transfer to the front axle that occurs during braking under driving conditions.
- It takes into account the aerodynamic effects.
- It can check the anti-lock braking system (ABS) that most of today’s cars have.
- Using a strain gauge, it would be a low-cost system.

This device for measuring braking force can be installed on any current vehicle equipped with disc brakes. The resulting wheel braking force can be used for various purposes:

- The braking torque can be displayed in real time to the driver of the vehicle so that he can conveniently adjust the force exerted on the brake pedal. This could be especially useful in the field of driver training.
- The braking torque obtained could also be used to improve the performance of existing brake control systems such as ABS, ASR, etc.

## Figures and Tables

**Table 1:** Strain gauge characteristics.

Parameters	Value
Type	FLA-3-11-1L
Gauge length	3 mm
Gauge factor	212 ± 1%
Gauge resistance	1203 ± 0.5
Compensation temperature	11 × 10^−6^/°C
Transversal sensitivity	0.3%

**Table 2:** Parameters.

Parameters	Value
Diameter of the hydraulic cylinder (d)	54 mm
Angle covered by the pad (α)	0.785 rad
External radius of pad (*R_e_*)	150 mm
Internal radius of pad (*R_i_*)	85 mm
